# Differential regulation of taurine biosynthesis in rainbow trout and Japanese flounder

**DOI:** 10.1038/srep21231

**Published:** 2016-02-16

**Authors:** Xuan Wang, Gen He, Kangsen Mai, Wei Xu, Huihui Zhou

**Affiliations:** 1 Key laboratory of Aquaculture Nutrition (Ministry of Agriculture), Ocean University of China, Qingdao 266003, PR China

## Abstract

Animals have varied taurine biosynthesis capability, which was determined by activities of key enzymes including cysteine dioxygenase (CDO) and cysteine sulfinate decarboxylase (CSD). However, whether CDO and CSD are differentially regulated across species remains unexplored. In the present study, we examined the regulations of CDO and CSD in rainbow trout and Japanese flounder, the two fish species with high and low taurine biosynthesis ability respectively. Our results showed that the expression of CDO was lower in rainbow trout but more responsive to cysteine stimulation compared to that in Japanese flounder. On the other hand, both the expression and catalytic efficiency (*k*_*cat*_) of CSD were higher in rainbow trout than those of Japanese flounder. A three-residue substrate recognition motif in rainbow trout CSD with sequence of F_126_/S_146_/Y_148_ was identified to be responsible for high *k*_*cat*_, while that with sequence of F_88_/N_108_/F_110_ in Japanese flounder led to low *k*_*cat*_, as suggested by site-directed mutagenesis studies. In summary, our results determined new aspects of taurine biosynthesis regulation across species.

Taurine (2-amino ethanesulfonic acid) is one of the most abundant free amino acids in vertebrates^1^. It plays important roles in multiple physiological processes including bile salt conjugation^2^, osmoregulation^3^, calcium homeostasis^4^, and trophism in the development of central nervous system^5^. Taurine is synthesized endogenously from sulfur amino acids such as methionine and cysteine. The major pathway of taurine *de novo* synthesis involves the sequential oxidation of cysteine to cysteine sulfinic acid (CSA) by cysteine dioxygenase (CDO, EC 1.13.11.20), decarboxylation by cysteine sulfinate decarboxylase (CSD, EC 4.1.1.29), and oxidation of the resulting hypotaurine to taurine by a putative hypotaurine dehydrogenase, which remains uncharacterized^6^,^7^. In this metabolic pathway, CDO and CSD has been characterized as the key enzymes that determine taurine biosynthesis capability^8^. Factors that influence taurine biosynthesis enzymes activities include hormone status^9^,^10^, development stages^11^, osmotic conditions^12^ and diet^13^,^14^.

Taurine biosynthesis ability varies greatly among species^15^,^16^. Livers from dog and rat have a high concentration of all enzymes required for taurine biosynthesis while those from man, monkey and cat exhibit extremely low activity of CSD^1^,^17^,^18^. A wide range of CSD activities was also obtained in different fish species^16^. Taurine biosynthesis is high in rainbow trout^19^ but low in Japanese flounder^20^ and turbot^21^. As a result, low biosynthesis ability makes taurine an essential nutrient for many species. In cats, many defects associated with taurine deficiency have been observed, such as retinal degeneration^22^, impairment of reproduction^23^, abnormal development^24^ and dilated cardiomyopathy^25^. Meanwhile, dietary taurine supplementation stimulated growth on multiple fish species, such as rainbow trout^26^, Japanese flounder^27^, turbot^21^, cobia^28^, and yellowtail^29^. In addition, taurine supplementation improved metamorphosis of *Solea senegalensis* larvae^30^.

To date, the differential taurine biosynthesis across species has been largely attributed to the activities of CDO and CSD enzymes, but the exact underlying mechanism has not been explored. Dietary sulfur amino acids stimulated taurine biosynthesis in rainbow trout^19^ but not in Japanese flounder^20^. Our previous study suggested that the response of CDO activities to dietary sulfur amino acids was less sensitive in turbot than that in mammals^21^. These results provide clues that the taurine biosynthesis might be differentially regulated among species.

Rainbow trout and Japanese flounder are teleost with high and low taurine biosynthesis respectively in spite of the similar zoological status and feeding habits^16^, therefore can serve as good model for comparative taurine biosynthesis studies across species. In the present study, the primary sequences of CDO and CSD in these species were identified. The expression and activities of CDO and CSD in fish livers were determined. The responses of CDO to cysteine stimulation were characterized. The kinetics of recombinant CDO and CSD proteins were also investigated.

## Results

### cDNA Cloning of CDO and CSD in rainbow trout and Japanese flounder

**Figure 1:**
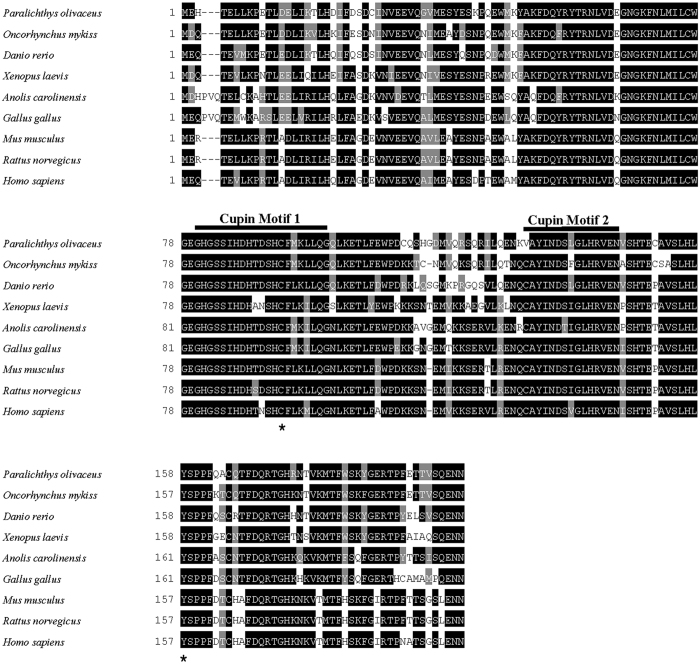
Multiple sequence alignment of CDOs. Highly conserved residues are indicated with black backgrounds and similar residues are shaded. The cupin superfamily motifs are indicated in the top line. Dashes indicate gaps. Asterisks show highly conserved cysteine and tyrosine residues formation of cross-linked cofactor of CDO. From top to bottom, the sequences are from *Paralichthys olivaceus* (KP739882), *Oncorhynchus mykiss* (KP739883), *Danio rerio* (Q6NWZ9), *Mus musculus* (NP_149026), *Rattus norvegicus* (AAH70509), *Homo sapiens* (AAH24241), and *Xenopus laevis* (NP_001083506)

In the present study, the full-length cDNAs of CDO and CSD from rainbow trout and Japanese flounder were cloned. The full-length cDNA of rainbow trout CDO was 817 bp, with an open reading frame (ORF) of 600 bp encoding 200 amino acids (GenBank Accession No. KP739883). The full-length cDNA of Japanese flounder CDO was 747 bp, with an ORF of 603 bp encoding 201 amino acids (GenBank Accession No. KP739882). The CDO amino acid sequence between rainbow trout (*Oncorhynchus mykiss*) and Japanese flounder (*Paralichthys olivaceus*) shared 84% identity. Multiple sequence alignment was done to compare CDO sequences across species, including rainbow trout (*Oncorhynchus mykiss*), Japanese flounder (*Paralichthys olivaceus*), zebrafish (*Danio rerio*, Q6NWZ9), amphibian (*Xenopus laevis*, NP_001083506), sauropsida (*Anolis carolinensis*, XP_003223077), chicken (*Gallus gallus*, XP_424964), mouse (*Mus musculus*, NP_149026), Rat (*Rattus norvegicus*, AAH70509), and human (*Homo sapiens*, AAH24241). As shown in Fig. 1, the primary sequence of CDO showed high homology across species. All CDO orthologs from various species contained two cupin motifs. The residues making of Cys-Tyr cofactor, a unique post-translational modification of CDO, are conserved in all species.

**Figure 2:**
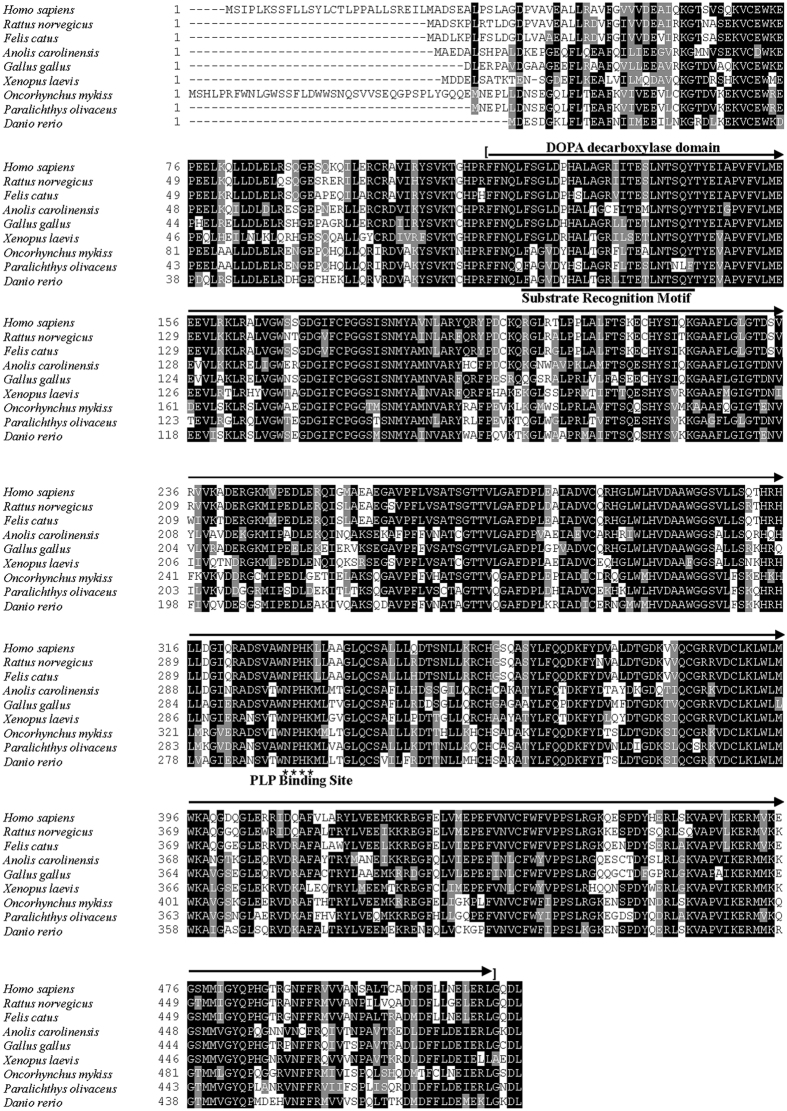
Multiple sequence alignment of CSDs. Highly conserved residues are indicated with black backgrounds and similar residues are shaded. Dashes indicate gaps. The conserved DOPA decarboxylase domain are indicated in the top line. The substrate recognition motif are indicated in the bottom line. Asterisks show the conserved residues in PLP binding site. From top to bottom, the sequences are from *Paralichthys olivaceus* (KP739884), *Takifugu rubripes* (ABF22453), *Danio rerio* (NP_001007349), *Cyprinus carpio* (BAE73113), *Oncorhynchus mykiss* (KP739885), *Mus musculus* (NP_659191), *Rattus norvegicus* (NP_068518), and *Homo sapiens* (NP_057073)

**Figure 3:**
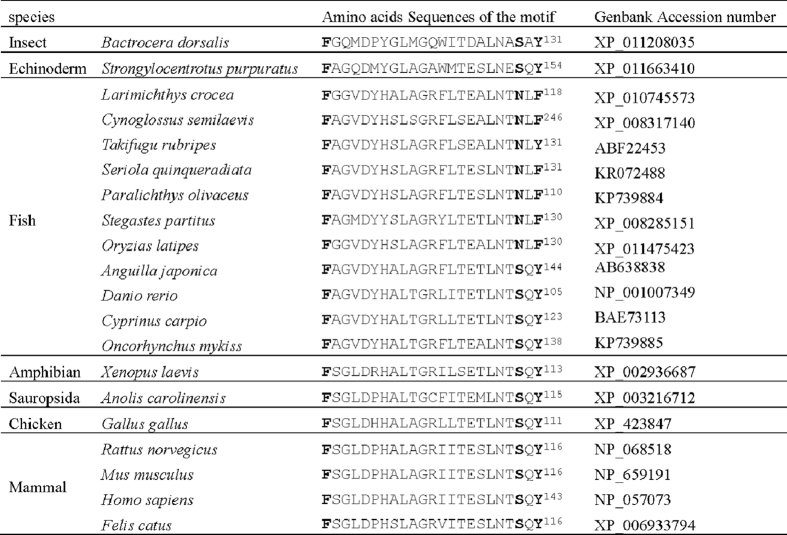
Sequence alignment of the substrate recognition motif in CSD across species. The three key residues in motif are bold. From top to bottom, the sequences are from insect, echinoderm, fish, amphibian, sauropsida, chicken and mammals

The full-length cDNAs of CSD isolated from rainbow trout and Japanese flounder were 1942 bp and 1751 bp respectively. The ORFs of rainbow trout and Japanese flounder CSD were 1575 bp encoding 525 amino acids (GenBank Accession No. KP739885) and 1461 bp encoding 487 amino acids (GenBank Accession No. KP739884) respectively. The predicted amino acid sequence of rainbow trout and Japanese flounder CSD shared 79% identity. Multiple alignment of amino acid sequences of CSD was conducted across species including rainbow trout, Japanese flounder, zebrafish (*Danio rerio,* NP_001007349), amphibian (*Xenopus laevis*, XP_002936687), sauropsida (*Anolis carolinensis*, XP_003216712), chicken (*Gallus gallus*, XP_423847), rat (*Rattus norvegicus*, NP_068518), cat (*Felis catus*, XP_006933794) and human (*Homo sapiens*, NP_057073). As shown in Fig. 2, a conserved enzymatic domain of DOPA decarboxylase and a pyridoxal phosphate (PLP) binding sequence (Asn-Pro-His-Lys, NPHK) were found in all species. The N-terminal region of CSD varied across species. As a member of the aspartate aminotransferase fold type I superfamily, a three-residue substrate recognition motif was present in CSD (Fig. 2). The three-residue F/S/Y motif was conserved in most species, including rainbow trout (F126, S146 and Y148). However, this motif was presented as F/N/F in Japanese flounder (F88, N108 and F110), yellowtail (*Seriola quinqueradiata*), medaka (*Oryzias latipes*), large yellow croaker (*Larimichthys crocea*), half-smooth tongue sole (*Cynoglossus semilaevis*), and bicolor damselfish (*Stegastes partitus*) (Fig. 3).

### Hepatic expression and activities of CDO and CSD in rainbow trout and Japanese flounder

**Figure 4:**
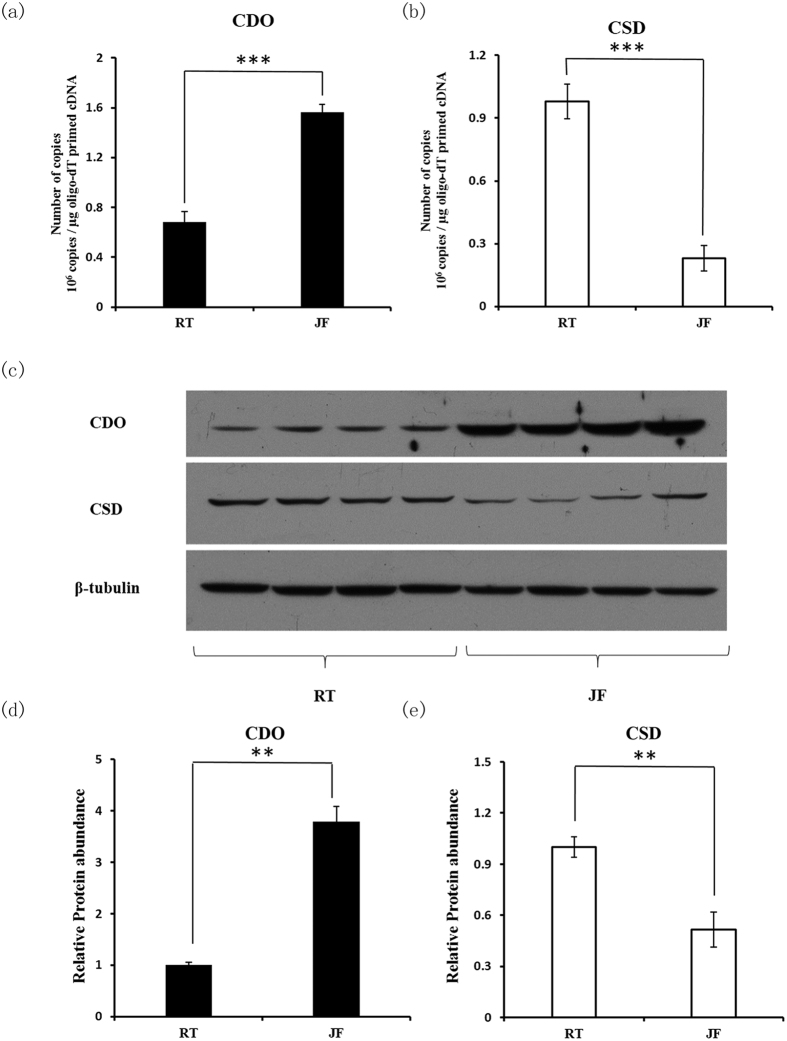
Hepatic expression of CDO and CSD in rainbow trout and Japanese flounder. Copy numbers of CDO and CSD in livers of rainbow trout (RT) and Japanese flounder (JF) were measured by a method of absolute quantification of cDNAs. The results are expressed as copies per μg oligo-dT primed cDNA and the data are presents as means ± S.E.M.(n = 6) (**a–b**). Protein abundance of CDO and CSD in fish livers was analysed using western blot. Aliquots of 20 μg protein was loaded in each line and β-tubulin was used as a loading control (**c**). The relative protein abundance of CDO and CSD in rainbow trout (RT) livers were normalized to the total protein loading and the expression levels in Japanese flounder (JF) were expressed as relative expression values to those in RT group. The data are expressed as means ± S.E.M.(n = 4) (**d,e**). The differences between experimental groups are tested using independent *t* tests. **p < 0.01, ***p < 0.001

A method for absolute quantification of cDNA using real-time PCR was used to compare the mRNA level of CDO and CSD in livers of rainbow trout and Japanese flounder^31^. As shown in Fig. 4, the copy number of CDO in per milligram oligo-dT primed cDNA of rainbow trout was 6.8 × 10^5^ while the value of Japanese flounder was 1.57 × 10^6^ (Fig. 4a). On the other hand, CSD mRNA levels in rainbow trout liver (9.8 × 10^5^ copies per mg cDNA) were significantly higher than that in Japanese flounder (2.3 × 10^5^ copies per mg cDNA, Fig. 4b).

The protein abundance of CDO and CSD in livers of rainbow trout and Japanese flounder were also detected (Fig. 4c–e). The relative protein level of CDO in rainbow trout was accounted for approximately 27% of that in Japanese flounder. However, the CSD protein level in rainbow trout was approximately 1.9 fold to that in Japanese flounder.

**Figure 5:**
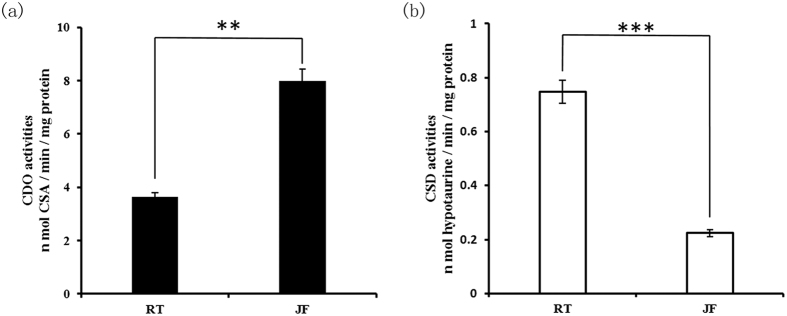
Enzyme activities of CDO and CSD in fish livers. The enzyme activities of CDO (**a**) are expressed as nmol CSA per min per mg protein and the enzyme activities of CSD (**b**) are expressed as nmol hypotaurine per min per mg protein. The data are expressed as means ± S.E.M.(n = 6). The differences between experimental groups of rainbow trout (RT) and Japanese flounder (JF) are tested using independent *t* tests. **p < 0.01; ***p < 0.001

The hepatic activities of CDO and CSD were also examined. As shown in Fig. 5a, the CDO activity in rainbow trout liver was 3.67 ± 0.15 nmol CSA/min/mg protein, which was signigicantly lower than that in Japanese flounder (7.99 ± 0.45 nmol CSA/min/mg protein). On the other hand, the hepatic CSD activity in rainbow trout (0.75 ± 0.04 nmol hypotaurine/min/mg protein) was approximately 3.3 fold to that in Japanese flounder (0.22 ± 0.01 nmol hypotaurine/min/mg protein, Fig. 5b).

### Differential regulation of cellular CDO expression

Upon cysteine stimulation, CDO forms the intramolecular Cys-Tyr cofactor (mature form), which appeared as a lower band in SDS-PAGE compared to the form without cofactor (immature form)^14^. The formation of Cys-Tyr cofactor could increase the catalytic efficiency by approximately 10 fold over the cofactor-free CDO^32^, which is critical in maintaining cysteine concentration below toxic levels^33^. In this study, fish CDOs were transfected into HepG2 cells, which contained no endogenous CDO activity^34^, thus provided a clean background for CDO activity measurement.

**Figure 6:**
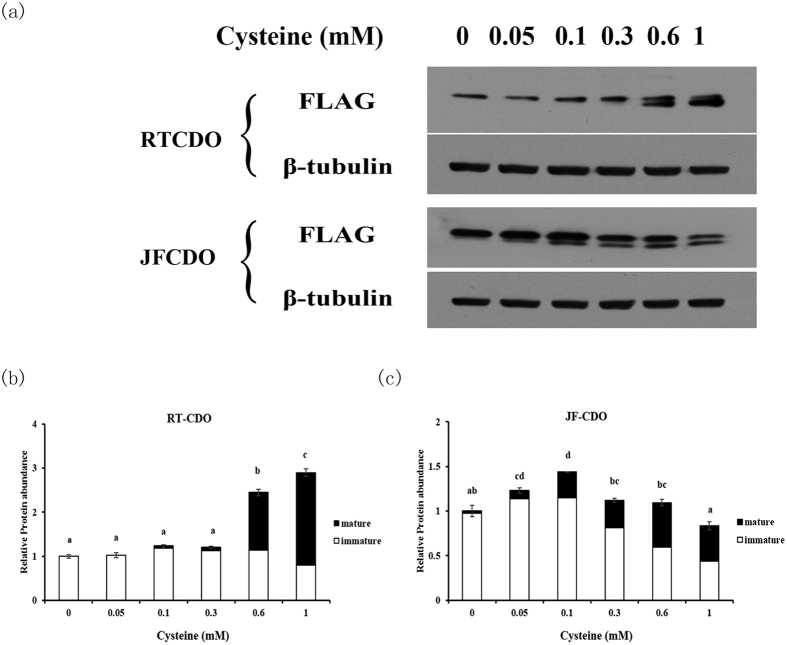
CDO expression in response to cysteine stimulation. Rainbow trout (RT) and Japanese flounder (JF) CDO were transfected into HepG2 cells and the expression in response to cysteine levels (0, 0.05, 0.1, 0.3, 0.6, 1 mM) was analyzed with western bolt. Aliquots of 20 μg protein was loaded in each line and β-tubulin was used as the loading control. The overexpressed CDO was detected with antibody against FLAG (**a**). CDO with Cys-Tyr cofactor (mature form, the lower band) and the cofactor-free form (immature form, the upper band) were quantified using NIH Image 1.63 software respectively. Relative protein abundance was expressed as relative expression value to the cysteine-free group (**b–c**). The data are expressed as means ± S.E.M. (n = 3). At least triplicates were conducted for each data point. The different expression of total CDO in response to cysteine were tested using one-way ANOVA and Tukey’s multiple-range test. Different letters above the bars denote significant differences between groups at the *p* < 0.05 level

Both fish CDOs showed responses after cells were treated with serial cysteine levels in medium. As shown in Fig. 6a, mature form of CDO with Cys-Tyr cofactor (shown as the lower band in SDS-PAGE) induced by cysteine was observed. However, CDO expression among species was differentially regulated. The protein levels of rainbow trout CDO increased 2.9 fold when cysteine levels were increased from 0 to 1 mM (Fig. 6b). On the contrast, the Japanese flounder CDO increased 1.4 fold when cysteine levels were increased from 0 to 0.1 mM and then decreased when cysteine levels from 0.1 to 1 mM (Fig. 6c). More importantly, the changes of CDO expression were mainly attributed to the changes of mature form of CDO with cofactor while the immature form were much more constant. When the concentration of cysteine increased to 1 mM, rainbow trout CDO was mainly expressed as the mature form (~73%) while the ratio of CDO mature form in Japanese flounder was approximately 48% (Fig. 6b,c).

### Kinetic characterization of CDO and CSD

**Figure 7:**
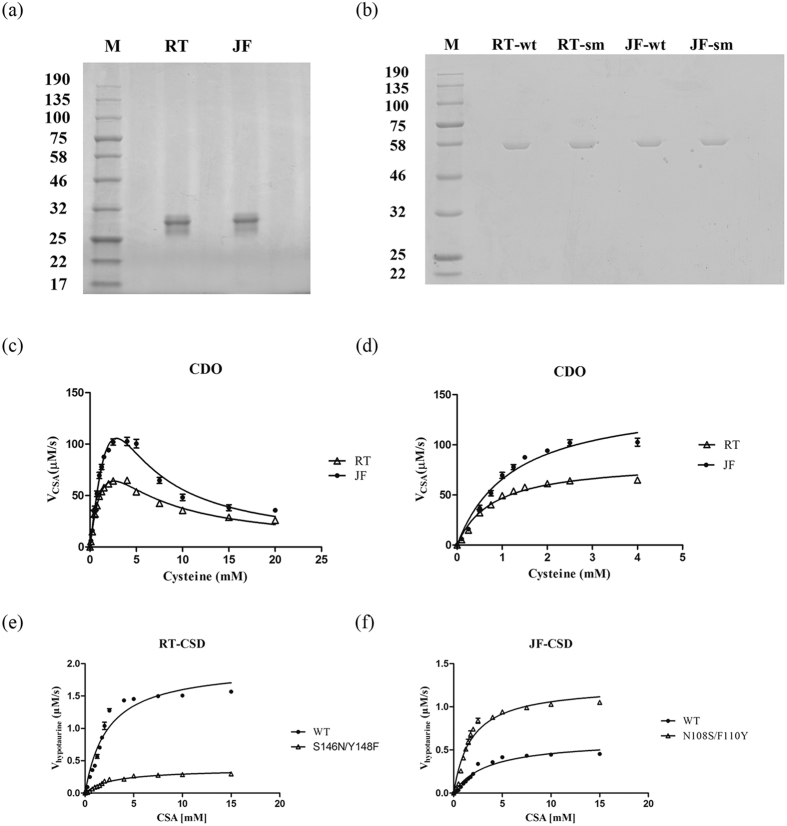
Kinetic characterization of recombinant CDO and CSD proteins. Recombinant rainbow trout (RT) and japanese flounder (JF) CDO were separated by 4-20% TruPAGE^TM^ Precast Gels (**a**). The wild-type (wt) and site-mutants (sm) of CSD proteins were separated by 10% SDS-PAGE gel (**b**). Activity of recombinant rainbow trout (RT) CDO and Japanses flounder (JF) CDO (0.2uM) was investigated using a wide substrate range (0-20 mM), which revealed a two phase kinetics (**c**). The kinetic parameters of fish CDOs were analyzed according to Michaelis-Menten results of CDO activities at cysteine concentrations below 4 mM (**d**). Activity of recombinant fish CSDs (0.5uM) at a CSA concentration of 0–15 mM were measured and it was with well-behaved Michaelis-Menten kinetics. Kinetic parameters of the wild type (WT) and the S146N/Y148F mutant of CSD from rainbow trout were analyzed according to Michaelis-Menten results (**e**). The kinetic parameters of the wild type (WT) and the N108S/F110Y mutant of CSD from Japanese flounder were analyzed according to Michaelis-Menten results (**f**). The kinetic characterization of recombinant proteins were estimated using nonlinear fitting by Prism 5 software (Graphpad software). The data are expressed as means ± S.E.M. (n = 3)

Both CDO and CSD proteins from rainbow trout and Japanese flounder were recombinantly expressed in *E.coli* and purified (Fig. 7a,b). The enzyme kinetics of CDO was determined using a wide range of substrate concentration (0–20 mM cysteine, Fig. 7c). Our result demonstraed a two phase CDO kinetics, a Michaelis-Menten model at low cysteine concentration (0–4 mM, Fig. 7d), and a substrate inhibition model at high cysteine concentration (>4 mM). The *K*_*m*_ of rainbow trout CDO and Japanese flounder CDO for cysteine was 0.79 ± 0.09 mM and 1.23 ± 0.15 mM respectively. The value of *k*_*cat*_ of CDO was 16.72 ± 0.67 s^−1^ in rainbow trout and 29.36 ± 1.52 s^−1^ in Japanese flounder.

The CSD activity was measured using a CSA concentration of 0–15 mM. As shown in Fig. 7e,f, according to the Michaelis-Menten equation, the *K*_*m*_ of rainbow trout and Japanese flounder CSDs were 2.30 ± 0.24 mM and 3.45 ± 0.38 mM respectively. The value of *k*_*cat*_ of CSD in rainbow trout and Japanese flounder was 4.72 ± 0.36 s^−1^ and 1.52 ± 0.13 s^−1^ respectively.

Sequences alignment analysis showed that the key residues within the active sites of fish CSDs were not well conserved, F_126_/S_146_/Y_148_ in rainbow trout versus F_88_/N_108_/F_110_ in Japanese flounder. In order to explore their impact on CSD catalytic activity, these key residues were switched between the two fishes by site-directed mutagenesis. The two CSD variants were also recombinantly expressed, purified and kinetic characterized. The *K*_*m*_ of rainbow trout CSD mutant (S146N/Y148F) was 2.73 ± 0.27 mM and the *k*_*cat*_was 1.86 ± 0.07 s^−1^ (Fig. 7e), which were similar to those of the wild-type Japanese flounder CSD. The *K*_*m*_ of Japanese flounder CSD mutant (N108S/F110Y) was 1.83 ± 0.17 mM and its *k*_*cat*_was 6.26 ± 0.20 s^−1^ (Fig. 7f), which were similar to those of the wild-type rainbow trout CSD.

## Discussion

Taurine biosynthesis is controlled by two key enzymes, CDO and CSD^8^. To date, CDO and CSD have been identified and characterized from mammals to eubacteria^32^,^35^,^36^,^37^,^38^, suggesting the taurine biosynthesis pathway via CDO and CSD is highly conserved during evolution. Given the fact that taurine biosynthesis ability varies greatly among species, it will be interesting to examine whether the regulation of CDO and CSD can be different among species. In the present study, we examined CDO and CSD from rainbow trout and Japanese flounder, two fish species with high and low taurine biosynthesis ability respectively in spite of the similar zoological status and feeding habits^16^.

We firstly characterized the full primary sequence of both CDO and CSD from rainbow trout and Japanese flounder. CDO is a member of the cupin superfamily composed of a wide range of functional proteins with two conserved cupin motifs for metal binding^39^,^40^. Both cupin motifs were identified in CDOs of rainbow trout and Japanese flounder (Fig. 1). In addition, the Cys-Tyr residues for intra-molecular crosslink^41^,^42^ were also highly conserved. CSD is a member of the group II pyridoxal 5′ - phosphate (PLP)-dependent amino acid decarboxylases family^43^,^44^. The DOPA decarboxylase domain and the NPHK sequence motif^35^ were both conserved in CSDs of rainbow trout and Japanese flounder.

Previously, taurine biosynthesis ability was only attributed to the activities of CDO and CSD^13^,^45^. Here we showed a far more complicated mechanism. Rainbow trout is low in CDO but high in CSD when compared with Japanese flounder, suggesting that CDO and CSD might play differential roles in regulation of taurine biosynthesis. It is known that CDO and CSD were differentially regulated by sulfur amino acids. High levels of dietary sulfur amino acids could markedly increase CDO activities and stimulated taurine biosynthesis^46^, while decreased CSD activities^47^. It remains to be investigated whether the different levels of CDO and CSD between rainbow trout and Japanese flounder is the result of evolutional adaptation for diets and osmotic conditions, both of which regulating CDO and CSD levels^12^,^46^,^47^.

CDO is known as one of the most highly regulated metabolic enzymes responding to sulfur amino acids^33^. Its concentrations increase robustly with elevated cysteine levels in diet^47^. However, this response seems to be different among species^21^. In this study, the differential regulations of fish CDOs exogenously expressed in HepG2 cells were examined. The response of rainbow trout CDO to cysteine stimulation increased ~2.9 fold when cysteine levels increased from 0 to 1 mM. This was much more dramatic than that of Japanese flounder (~1.4 fold). An analogous study showed that rat CDO exogenously expressed in HepG2/C3A cells increased 1.6 fold upon the same stimulation^34^. It is known that CDO concentrations are regulated by cysteine levels via the formation of intramolecular Cys-Tyr cofactor, which could dramatically increase CDO’s catalytic efficiency^14^,^48^ and stability^49^. In this study, the crosslink formation efficiency between the two fishes was different (~73% crosslinked form CDO in rainbow trout while only ~48% in Japanese flounder at 1 mM cysteine). To date, several residues in the active site of CDO that affect the cofactor formation have been characterized^14^,^41^,^42^,^48^,^50^,^51^. Cys-93 and Tyr-157 are involved in cofactor formation directly; His-86, His-88 and His-140 are vital for iron coordination; Arg-60 is the only charged residue in the active site and probably plays a role in hydrogen bonding with cysteine. However, sequence alignment showed that all the previously identified residues in CDO active site were conserved in both rainbow trout and Japanese flounder. The reason for the differential regulation of cofactor formation among species remains to be further explored.

Due to the profound influence of cofactor formation on CDO catalytic efficiency, recombinant CDO proteins and kinetic assays in this study were performed under conditions with iron and oxygen, which favored cofactor formation^14^. The two fish CDOs showed comparable kinetic parameters, which might be a result of the high identities (84%) of the deduced amino acids sequences. The *K*_*m*_ of 0.79 mM for rainbow trout CDO and 1.23 mM for Japanese flounder CDO are in line with the reported kinetic parameters of human (*K*_*m*_ = 0.77 mM)^48^ and rat (*K*_*m*_ = 0.45 mM)^51^. It is known that CDO plays a critical role in regulation of cysteine homeostasis^33^. The similarity CDO kinetic parameters among species indicate that maintaining cysteine concentration below the toxic level are important for the living organism. However, Ye and colleagues reported a *K*_*m*_ of 3.1 mM for human CDO^50^, a result probably caused by differential enzyme preparations^14^,^50^. In addition, our results confirmed CDO was inhibited at high substrate concentrations, similar to what was reported for rat CDO and human CDO^14^,^48^,^51^. Crystal structure studies showed that Cys-164 lined in the only access of substrate to the active site. When cysteine concentrations was high (millimolar levels), cysteine could form a disulfide with Cys-164 and inhibited CDO catalysis^51^.

Despite of the high identities (79%) of the deduced amino acids sequences of CSD, kinetic characterization of fish CSDs showed a great difference of catalytic efficiency. The *k*_*cat*_ of rainbow trout CSD (4.72 ± 0.36 s^−1^) was about 3.1 fold of that in Japanese flounder CSD (1.52 ± 0.13 s^−1^). Recently, a three residue substrate recognition motif (Faa_19_SaaY) has been identified in the active site of human CSD and was shown to dramatically affect the catalytic efficiency^32^. Sequence alignment of the motif showed that the residues, especially the latter two residues, were less conserved in fishes (Fig. 3). The motif was presented as F/S/Y in rainbow trout but as F/N/F in Japanese flounder and yellowtail^52^, which also showed a limited taurine biosynthesis capability^29^. In order to explore the impact of this motif variation on CSD catalytic activity, the key residues within the two fish CSDs were switched. Results of the kinetic characterization showed that the *k*_*cat*_ of rainbow trout S146N/Y148F variant (1.86 ± 0.07 s^−1^) was similar to that of wild-type Japanese flounder CSD, while an analogous result was obtained in Japanese flounder. It indicates that the F/N/F motif in Japanese flounder CSD was responsible for the low catalytic activity and the limited taurine biosynthesis capability. Structural analyses of human CSD (PDB: 2JIS) showed that the phenolic side chain at X_3_ position of the F/S/Y motif was crucial for CSA binding, which might form a hydrogen bond with the lone pair of electrons on the sulfur atom of CSA^32^. In addition, the F/N/F motif was also present in many other fishes with unknown taurine biosynthesis capability, such as medaka^52^, large yellow croaker, half-smooth tongue sole and bicolor damselfish. Their ability of taurine biosynthesis remains to be examined.

In conclusion, our results identified the differential taurine biosynthesis ability between rainbow trout and Japanese flounder and determined the regulation of taurine biosynthesis was a far more sophisticated system than ever thought before.

## Materials and Methods

### Tissue collection

Rainbow trout (76.4 ± 5.8 g) and Japanese flounder (98.7 ± 7.2 g) at the same age (4 months old) were obtained from local rearing farms (Weifang and Yantai respectively, China). After overnight fasting to remove possible nutritional influences on taurine biosynthesis^53^,^54^, fish was anesthetized with 3-aminobenzoic acid ethyl ester (MS222) at 100 μg/ml. Liver, the major site of taurine biosynthesis, was rapidly removed and immediately frozen in liquid nitrogen and stored at −80 °C before use. All procedures performed in study were in strict accordance with the recommendations in the Guide for the Use of Experimental Animals of Ocean University of China. The protocols for animal care and handing used in this study were approved by the Institutional Animal Care and Use Committee of Ocean University of China.

### Molecular cloning and characterization of full length cDNAs

Total liver RNA was isolated using TRizol reagent (Invitrogen). The quality and concentration of RNA were measured by Nanodrop 2000 (Thermo Fisher Scientific). First strand cDNA was produced by SMART RACE cDNA Amplification Kit (Clontech) and used as template for 3′ or 5′ rapid amplification of cDNA ends (RACE) PCR. To obtain the full length cDNAs of CDO and CSD, degenerative primers were designed on the conserved regions from different species using CODEHOP online software (http://blocks.fhcrc.org/blocks/codehop.html) firstly. Subsequently, specific primers were further designed for RACE PCR with Primer 5 software. All PCR products were separated by agarose gel and the target band was purified, and then ligated into pEASY-T1 vector (Transgen Biotech). The PCR products were sequenced in Sangon Biotech Co., Ltd (Shanghai). The full-length cDNA sequences were deposited to the GenBank database. All the primers used in this study were listed in Table 1.

### Absolute mRNA quantification

In order to compare the mRNA levels of CDO and CSD in livers of juvenile rainbow trout and Japanese flounder, absolute quantification of mRNA using quantitative real-time PCR (qRT-PCR) was conducted as described by Whelan *et al.* (2003)^31^. Briefly, the PCR products, obtained from real-time PCR primers for specific target gene, were cloned into the pEASY-T1 vector. The plasmid was further transformed into DH5α competent cells (Takara). Single colonies were grown up in LB-broth medium and plasmid was subsequently purified using the SanPrep Column Plasmid Mini-Preps Kit (Sangon Biotech). Sequence verified plasmids were serially diluted and used for plasmid standards after linearized by *Hind* III (New England Biolabs). Single-stranded cDNA from fish tissues was synthesized using a PrimeScript^®^ RT Reagent Kit (Takara) and the concentration of cDNA was measured by Nanodrop 2000 (Thermo Fisher Scientific).

The qRT-PCR was carried out in a quantitative thermal cycler Mastercycler ep realplex (Eppendorf). SYBR Green real-time PCR kit (Takara) was used. The melting curve was performed after the amplification phase for confirmation the specificity of production. The plasmid standards and tissue cDNA samples were detected in a same plate and each sample was run in triplicate. Target gene expression levels were quantitated as copy number per microgram of oligo-dT primed cDNA according to the curves of plasmid standards.

### Recombinant Protein expression and purification

For the purpose of producing recombinant protein in bacterial expression systems, the open reading frame (ORF) of CDO and CSD from rainbow trout and Japanese flounder were subcloned into the pET-28a expression plasmid (Novagen) using *BamH*I and *Hind*III restriction sites. Sequence verified plasmid was transformed into *E.coli* BL21 (DE3) competent cells (Transgen Biotech) for protein expression.

In order to explore the impact of the residues within the active sites on CSD catalytic activity, two double-sties CSD variants in the pET-28a vector were constructed by the QuikChange Lightning Site-Directed Mutagenesis kit (Stratagene). The S146 and Y148 residues in rainbow trout CSD were mutated to N and F, while the N108, and F110 residues in Japanese flounder CSD were replaced to S and Y, respectively. The mutant expression constructs were sequence verified and transformed into *E.coli* BL21 (DE3) competent cells for protein expression.

Transformed cells were cultured in LB medium supplemented with 50 μg/mL kanamycin at 37 °C. Expression of CDO was induced with 0.2 mM isopropyl β-D-1-thiogalactopyranoside (IPTG) at OD_600_ = 0.5, and continued for 4 hours at 37 °C. The cell were harvested by centrifugation at 6,000 g for 20 min at 4 °C. Expression of CSD was induced at OD_600_ = 0.6 with 0.5 mM IPTG at 25 °C overnight.

All expressed proteins carried an N-terminal 6 × His-tag and were purified using HisTrap HP columns (GE Healthcare) on an ÄKTA FPLC Purifier system (Amersham Biosciences). Due to the loose binding of iron in the active center of CDO, expression media and all purification buffers of CDO were supplemented with 1 mM ammonium iron sulfate to ensure proper iron saturation^48^. The cell pellets were resuspended in cold lysis buffer (10 mM NaH_2_PO_4_, 300 mM NaCl, 20 mM imidazole, pH 8.0). The cell suspensions were kept in an ice-water bath and sonicated (Sonics, VC130) followed by centrifugation at 12000 g for 20 min at 4 °C. The supernatants were filtered using 0.45 μm pore membranes and applied to the HisTrap HP columns. Columns were washed using a gradient elution with Buffer A (10 mM NaH_2_PO_4_, 300 mM NaCl, 20 mM imidazole, pH 8.0) and Buffer B (10 mM NaH_2_PO_4_, 300 mM NaCl, 500 mM imidazole, pH 8.0). Protein elution fractions were further purified using a Superdex 200 column (GE Healthcare) with activity buffers (CDO buffer: 100 mM NaCl, 20 mM Tris, pH 8.0; CSD buffer: 100 mM NaH_2_PO_4_,10 μM pyridoxal 5′ - phosphate, pH 7.4). The purified protein samples were concentrated ~10 fold using Amicon ultra 10 K filters (Millipore). Purified protein samples were stored at −80 °C.

### Enzyme assays

Activity assays for tissue samples and recombinant CDO proteins were done as described previously^55^. For tissues, samples were homogenized in 50 mM 2-(N-Morpholino) ethanesulfonic acid (MES) buffer (pH 6.1) with a homogenizer and centrifuged at 25,000 g for 30 min at 4 °C. The supernatant was used for CDO enzyme assay. The activity assay was conducted in a total volume of 400 μl containing the following components: 62.5 mM MES buffer, 0.3 mM ferrous sulfate, 2 mM NAD, 5 mM hydroxylamine, 5 mM cysteine, and 62.5 μM bathocuproine disulfonate. For purified recombinant CDO proteins, the reaction mixture contained 0.2 μM purified CDO, 62.5 mM MES buffer, 0.3 mM ferrous sulfate, 62.5 μM bathocuproine disulfonate and varying concentrations of L-cysteine (0–20 mM). The reaction was started by addition of cysteine and incubated at 37 °C with vigorous shaking at 900 rpm to ensure proper oxygenation using a thermomixer (Eppendorf). The reaction was terminated by the addition of 200 μl 5% sulfosalicylic acid and the reaction mixtures were placed on ice for 15 min. After centrifugation, the reaction product, CSA, was collected in supernatant and measured by HPLC (HP 1100 system, Agilent Technologies).

CSD activities in tissue samples and recombinant proteins were measured as described previously^16^,^32^,^56^. Briefly, tissue samples were homogenized in 50 mM phosphate buffer (pH 6.8). The homogenates were centrifuged at 21,000 g for 15 min at 4 °C. The supernatant was used for CSD enzyme assay. The activity assay mixture (0.5 ml final volume) contained 15 mM glutamate, 25 mM CSA, 0.8 mM pyridoxal 5′ - phosphate (PLP), 0.55 mM DTT and 0.1 ml tissue homogenates. After incubation at 37 °C for 30 min, the reaction was terminated by 10% trichloroacetic acid. For recombinant CSD proteins, enzymatic assays were conducted in a final volume of 0.2 ml containing 0.5 μM purified CSD, 100 mM NaH_2_PO_4_, 10 μM PLP and varying concentrations of CSA (0–15 mM). After incubation at 37 °C for 10 min, the reaction was terminated by 20 μl of 1 M hydrochloric acid. After centrifugation, the reaction product, hypotaurine, was measured by HPLC.

The products of enzyme reactions (CSA and hypotaurine) were pre-column derivatized with *o*-phthaladehyde (OPA)/2-mercaptoethanol and separated with a 4.6 × 250 mm Zorbax Eclipse C18 column (Agilent Technologies) using a gradient elution. The derivatized product was detected using a fluorescence detector (FLD). The gradient mobile phase and the parameters of FLD were set as described previously^55^,^56^.

### Cell culture, transfection, and treatment

For the transient expression of CDO protein in mammalian cells, the ORF of CDOs were subcloned into the pGen2.1 vector (GeneScript) using *Not*I and *BamH*I restriction sites. Sequence verified plasmid was transformed into DH5α competent cells (TakaRa) and purified by EndoFree Plasmid Maxi Kit (Qiagen). The human hepatoma cells (HepG2 cells) were obtained from the Type Culture Collection of the Chinese Academy of Sciences, Shanghai, China. Cells were maintained in DMEM (Invitrogen) supplemented with 10% FBS (Invitrogen), 1% penicillin-streptomycin, 2 mM GlutaMAX^TM^ and 1 mM sodium pyruvate in a humidified incubator at 37 °C and 5% CO_2_. Cells were transfected with pGen2.1-CDO constructs using lipofectamine 3000 (Invitrogen) according to the manufacturer’s instructions. Cells transfected with empty pGen2.1 vector were used as control. After 24 hrs, transfected cells were evenly splitted and treated with experimental mediums for 24 hrs before lysis. The experimental medium was made by supplementing sulfur amino acid-free DMEM with 10% FBS, 1% penicillin-streptomycin, 2 mM GlutaMAX^TM^, 1 mM sodium pyruvate, 0.2 mM L-methionine and varying concentrations of L-cysteine (0, 0.05, 0.1, 0.3, 0.6, or 1 mM). Bathocuproine disulfonate (0.05 mM) was added to reduce the rate of cysteine auto-oxidation to cystine. All cell culture experiments were repeated at least three times.

### Immunoblotting analysis

The protein abundance of CDO and CSD in livers of rainbow trout and Japanese flounder were quantified using western blotting. Polyclonal rabbit antibody against CDO (#C6247) was purchased from Sigma, and anti-β-tubulin (#2146) was purchased from Cell Signaling Technology. A rabbit polyclonal antiserum against CSD was generated as described previously^11^. Frozen tissue samples were homogenized in ice-cold RIPA buffer (50 mM Tris-HCl, pH 7.4, 150 mM NaCl, 1 mM EDTA, 0.5% Nonidet P-40, 0.1% SDS) with protease inhibitor cocktail (Roche Applied Science). The homogenate was centrifuged at 12000 g for 30 min at 4 °C. Protein concentrations in supernatant were determined by a BCA protein assay kit (Beyotime) using bovine serum albumin as a standard. Aliquots of 20 μg protein were subjected to 12% SDS-PAGE and transferred to PVDF membrane. The membrane was blocked with 5% nonfat milk in TBST buffer (20 mM Tris-HCl, 500 mM NaCl and 0.1% Tween 20) and incubated with primary antibody overnight at 4 °C. Horseradish peroxidase (HRP)-conjugated secondary antibodies were detected using ECL reagents (GoodHere Inc., China). The density of the protein bands was quantified using NIH Image 1.63 software.

The expression of CDO in response to cysteine levels was evaluated using monoclonal mouse antibody against FLAG (#A00187, GeneScript). Cells were harvested and lysed in ice-cold RIPA buffer supplemented with protease inhibitors. The lysates were centrifuged at 12,000 g for 20 min at 4 °C. The cleared supernatants were collected and stored at −80 °C. Protein concentrations were determined with a BCA protein assay kit. Aliquots of 20 μg of total protein was loaded and separated by 4–20% TruPAGE^TM^ Precast Gels (Sigma) and followed with western bolt analysis. β-Tubulin was used as the loading control. At least triplicates were conducted for each data point.

### Statistical analysis

Each value is expressed as means ± S.E.M. Statistical analysis of expression levels and activities of CDO and CSD between rainbow trout and Japanese flounder were performed by independent *t* tests. The different expression of CDO in response to cysteine stimulation were tested using one-way ANOVA and Tukey’s multiple-range test. The kinetic characterization of recombinant proteins were estimated using nonlinear fitting by Prism 5 software (Graphpad software). A value of *P* < 0.05 was considered statistically significant.

## Additional Information

**How to cite this article**: Wang, X. *et al.* Differential regulation of taurine biosynthesis in rainbow trout and Japanese flounder. *Sci. Rep.* **6**, 21231; doi: 10.1038/srep21231 (2016).

## Figures and Tables

**Table 1:** Sequences of the primers used in this study.

	Name	Primer sequence (5′ – 3′)	Description
Degenerative primers	CDO-F	RTYAATGTRGAGGAGGTSCA	Degenerate PCR
	CDO-R	YTRCTCCAGAASGTCATCTTG	Degenerate PCR
	CSD-F	GTCAYCCWCKGTTYTTCAA	Degenerate PCR
	CSD-R	TTGTCBCCCGTGTCCAGA	Degenerate PCR
Rainbow trout	RTCDO-race F	TCCGCTGGTCAAAGGTCTGG	RACE PCR
	RTCDO-race R	TCCGCTGGTCAAAGGTCTGG	RACE PCR
	RTCSD-race F	TGTATGGTCAGCAGGAAATG	RACE PCR
	RTCSD-race R	TCATTTCCTGCTGACCATA	RACE PCR
	RTCDO-rtF	TCAAGATGACGTTCTGGAGCAA	Real time PCR
	RTCDO-rtR	GGGCAGTGATAAAGCCATTCTA	Real time PCR
	RTCSD-rtF	ATGCCTTGACAGGACGATTC	Real time PCR
	RTCSD-rtR	GCCGCTTTCATCACAGAGTAG	Real time PCR
	RTCDO- BamH I F	CGCGGATCCATGGACCAAACCGAGCTGCT	Protein expression
	RTCDO- Hind III R	CCCAAGCTTTCATTAGTTATTTTCTTGTGAGA	Protein expression
	RTCSD- BamH I F	CGCGGATCCATGAGTCACCTACCTAGATT	Protein expression
	RTCSD- Hind III R	CCCAAGCTTTCATTACAAATCACTCCCAAGCC	Protein expression
	RTCDO- Not I F	TTGCGGCCGCTATGGACCAAACCGAGCTGCT	Cell transfection
	RTCDO- BamH I R	CGCGGATCCTCATTAGTTATTTTCTTGTGAGA	Cell transfection
	RTCSD-site mutant F	ACCTCGTAGGTAAACTGGTTAGTGTTAAGAGCCTCCGTAAG	Site-directed mutagenesis
	RTCSD-site mutant R	CTTACGGAGGCTCTTAACACTAACCAGTTTACCTACGAGGT	Site-directed mutagenesis
Japanese flounder	JFCDO-race F	CAGCAGCATCCACGACCACA	RACE PCR
	JFCDO-race R	CCCAGGGAGTCGTTTATGT	RACE PCR
	JFCSD-race F	TTGACCCTCTGGACCACATT	RACE PCR
	JFCSD-race R	AGGCTCCTTGAACTGTGGTG	RACE PCR
	JFCDO-rtF	AAGGAACTTGGTGGATGAGG	Real time PCR
	JFCDO-rtR	CCCAGGGAGTCGTTTATGTA	Real time PCR
	JFCSD-rtF	TTGACCCTCTGGACCACATT	Real time PCR
	JFCSD-rtR	GTGGCACTGCTTCAACAAAT	Real time PCR
	JFCDO- BamH I F	CGCGGATCCATGGAGCACACCGAGCTGCT	Protein expression
	JFCDO- Hind III R	CCCAAGCTTTCATCAGTTGTTCTCTTGGGAAA	Protein expression
	JFCSD- BamH I F	CGCGGATCCATGAATGAACCTCTCCTGGA	Protein expression
	JFCSD- Hind III R	CCGGAATTCTCACAAATCGTTTCCCAGTCTT	Protein expression
	JFCDO- Not I F	TTGCGGCCGCTATGGAGCACACCGAGCTGCT	Cell transfection
	JFCDO- BamH I R	CGCGGATCCTCAGTTGTTCTCTTGGGAAA	Cell transfection
	JFCSD-site mutant F	CCACCTCATAGGTGTAAAGGCTAGTGTTGAGCGACTCTGT	Site-directed mutagenesis
	JFCSD-site mutant R	ACAGAGTCGCTCAACACTAGCCTTTACACCTATGAGGTGG	Site-directed mutagenesis
